# Evolution of Our Understanding of XIAP Deficiency

**DOI:** 10.3389/fped.2021.660520

**Published:** 2021-06-17

**Authors:** Anne C. A. Mudde, Claire Booth, Rebecca A. Marsh

**Affiliations:** ^1^Molecular and Cellular Immunology Section, UCL Great Ormond Street Institute of Child Health, London, United Kingdom; ^2^Department of Immunology and Gene Therapy, Great Ormond Street Hospital, London, United Kingdom; ^3^Division of Bone Marrow Transplantation and Immune Deficiency, Cincinnati Children's Hospital Medical Center, Cincinnati, OH, United States; ^4^Department of Pediatrics, University of Cincinnati, Cincinnati, OH, United States

**Keywords:** XIAP deficiency, X-linked lymphoproliferative disease, haemophagocytic lymphohistiocytosis, inflammatory bowel disease, inflammasome, haematopoietic stem cell transplantation, NOD2, BIRC4

## Abstract

X-linked inhibitor of apoptosis (XIAP) deficiency is a rare inborn error of immunity first described in 2006. XIAP deficiency is characterised by immune dysregulation and a broad spectrum of clinical manifestations, including haemophagocytic lymphohistiocytosis (HLH), inflammatory bowel disease (IBD), hypogammaglobulinemia, susceptibility to infections, splenomegaly, cytopaenias, and other less common autoinflammatory phenomena. Since the first description of the disease, many XIAP deficient patients have been identified and our understanding of the disease has grown. Over 90 disease causing mutations have been described and more inflammatory disease manifestations, such as hepatitis, arthritis, and uveitis, are now well-recognised. Recently, following the introduction of reduced intensity conditioning (RIC), outcomes of allogeneic haematopoietic stem cell transplantation (HSCT), the only curative treatment option for XIAP deficiency, have improved. The pathophysiology of XIAP deficiency is not fully understood, however it is known that XIAP plays a role in both the innate and adaptive immune response and in immune regulation, most notably through modulation of tumour necrosis factor (TNF)-receptor signalling and regulation of NLRP3 inflammasome activity. In this review we will provide an up to date overview of both the clinical aspects and pathophysiology of XIAP deficiency.

## Introduction

X-linked inhibitor of apoptosis (XIAP) deficiency is a rare inborn error of immunity caused by mutations in the *XIAP/BIRC4* gene. The disease is estimated to occur in 1–2 per million live male births (1). XIAP deficiency was first described in 2006 and is associated with a variety of disease manifestations, including recurrent haemophagocytic lymphohistiocytosis (HLH), inflammatory bowel disease (IBD), hypogammaglobulinemia, severe and/or recurrent infections, splenomegaly, and cytopaenias (2–4). However, as more patients have been identified over the years, other disease manifestations are now well-described. Treatment generally consists of immunosuppression and, in severe cases, allogeneic haematopoietic stem cell transplantation (HSCT). The XIAP protein is believed to be involved in both the innate and adaptive immune response. Furthermore, XIAP has a role in regulation of inflammasome activity (3, 5, 6). However, the pathophysiology of XIAP deficiency remains to be fully comprehended. This review will provide an up to date summary of our understanding of XIAP deficiency.

## History

The *XIAP/BIRC4* gene was first characterised in 1996 (7–9). However, XIAP deficiency underlying a primary immunodeficiency disorder was not described until 2006, when Rigaud et al. (2) found pathogenic variants in *XIAP* in male patients from 3 families with X-linked lymphoproliferative syndrome (XLP) phenotypes who lacked *SH2D1A* mutations. Following this initial report, XIAP deficiency was classified as XLP-2, while signalling lymphocytic activation molecule (SLAM)-associated protein (SAP) deficiency was referred to as XLP-1. XIAP deficient patients were initially observed to suffer from similar symptoms to SAP deficient patients, including HLH that was frequently triggered by an EBV infection, splenomegaly, cytopaenias, and hypogammaglobulinemia (2, 10). However, over time it became clear that the clinical features of XIAP deficiency differ significantly from those observed in SAP deficiency. Most striking is the fact that XIAP deficient patients do not develop lymphomas (2, 3, 11). Additionally in XIAP deficiency, HLH generally has a milder disease course with a lower mortality rate, but occurs more frequently and is often recurrent (1, 3, 12–14). A significant number of XIAP patients suffer from colitis, a disease manifestation that is observed less frequently in SAP deficient patients (4, 11, 12, 15, 16). Underlying these distinct disease manifestations is a difference in disease pathophysiology. Contrary to SAP deficiency, T and NK cell-cytotoxicity responses are normal, including those specific for EBV, as are numbers of switched memory B cells (2, 3, 17). On a genetic level no relation between the *SH2D1A* and *XIAP/BIRC4* genes has been identified, despite the fact that the two genes are localised in close proximity of each other in Xq25 (12, 18). Subsequent clinical observations led to a proposal that XIAP deficiency more readily fit the classical phenotype of familial HLH (FHL) (12), however a significant number of XIAP deficient patients do not develop HLH (4, 11, 14). Today, XIAP deficiency is regarded primarily as a disorder of immune dysregulation and hyperinflammation.

## Genetics

**Figure 1:**
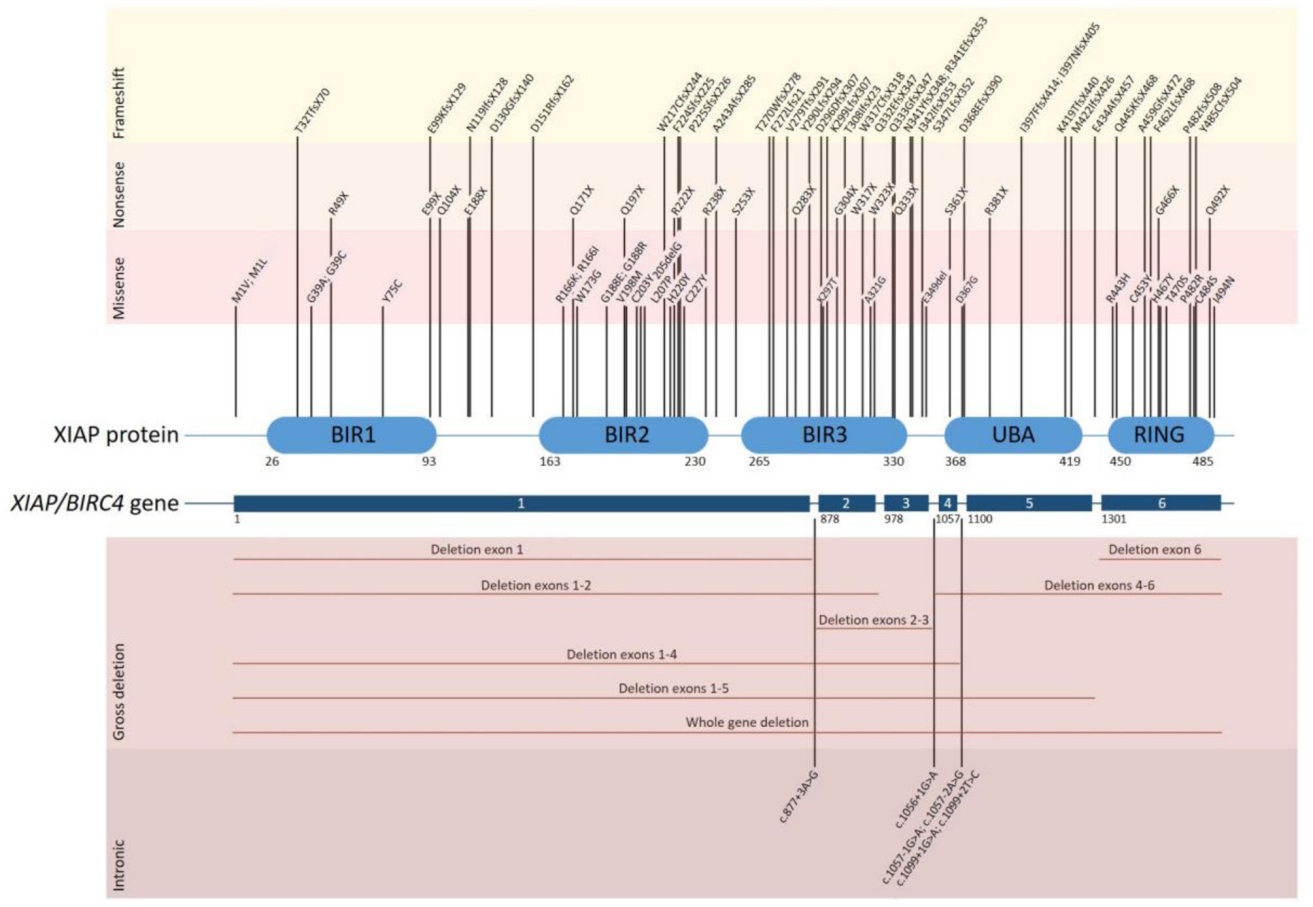
Overview of all disease causing mutations in the *XIAP/BIRC4* gene that have thus far been reported. The position of each of the reported mutations associated with XIAP deficiency is depicted along the XIAP protein and gene structure. Shown are the 6 coding exons that are present in the *XIAP/BIRC4* gene and the 5 functional domains of the XIAP protein. Mutations are grouped by mutation type. Missense mutations cluster in the BIR2 and RING domain of the XIAP protein.

XIAP is encoded by the *XIAP/BIRC4* gene, which consists of 6 coding exons. To date over 90 disease causing mutations have been described (Figure 1). Mutations are distributed along the length of the gene and include nonsense and missense mutations, large whole exon deletions, small insertions and deletions, often leading to a frameshift mutation, and intronic mutations. Nonsense mutations and deletions generally lead to absence of XIAP protein, whereas missense and splice site mutations may lead to residual expression of full-length or truncated, but non- or dysfunctional protein. Our updated overview of all known mutations corroborates the observation that missense mutations cluster in two hotspots that target either the BIR2 domain or the RING domain, highlighting the importance of these two domains in XIAP function (1, 5, 19). There is no clear correlation between genotype and phenotype, as shown by the large variability in clinical manifestations observed in affected siblings (4, 11, 20–22). Speckmann et al. (4) even found that neither the nature of the mutation, nor residual protein expression was correlated to clinical presentation. In contrast, Pachlopnik Schmid et al. found that XIAP deficient patients with null mutations more frequently developed HLH (14). It is likely that other genetic and environmental factors influence the clinical phenotype.

Female carriers of a *XIAP/BIRC4* mutation are generally asymptomatic. However, symptomatic female carriers have been described, expressing a variety of symptoms, including HLH-like disease, colitis and skin manifestations (21, 23, 24). Studies show that in the peripheral blood leukocytes of symptomatic female carriers, X-chromosome inactivation is either random or skewed to the mutant allele. In contrast, X-chromosome inactivation is skewed towards the wild type allele in asymptomatic female carriers, suggesting that cells expressing wild type XIAP have a selective survival advantage, possibly due to the anti-apoptotic activity of XIAP (2). Why certain female carriers have an abnormal inactivation pattern and whether the severity of the clinical presentation depends on the degree of X-inactivation and the corresponding residual expression of wild type XIAP protein and function in female carriers, remains to be discovered.

## XIAP Protein Structure and Function

**Figure 2:**
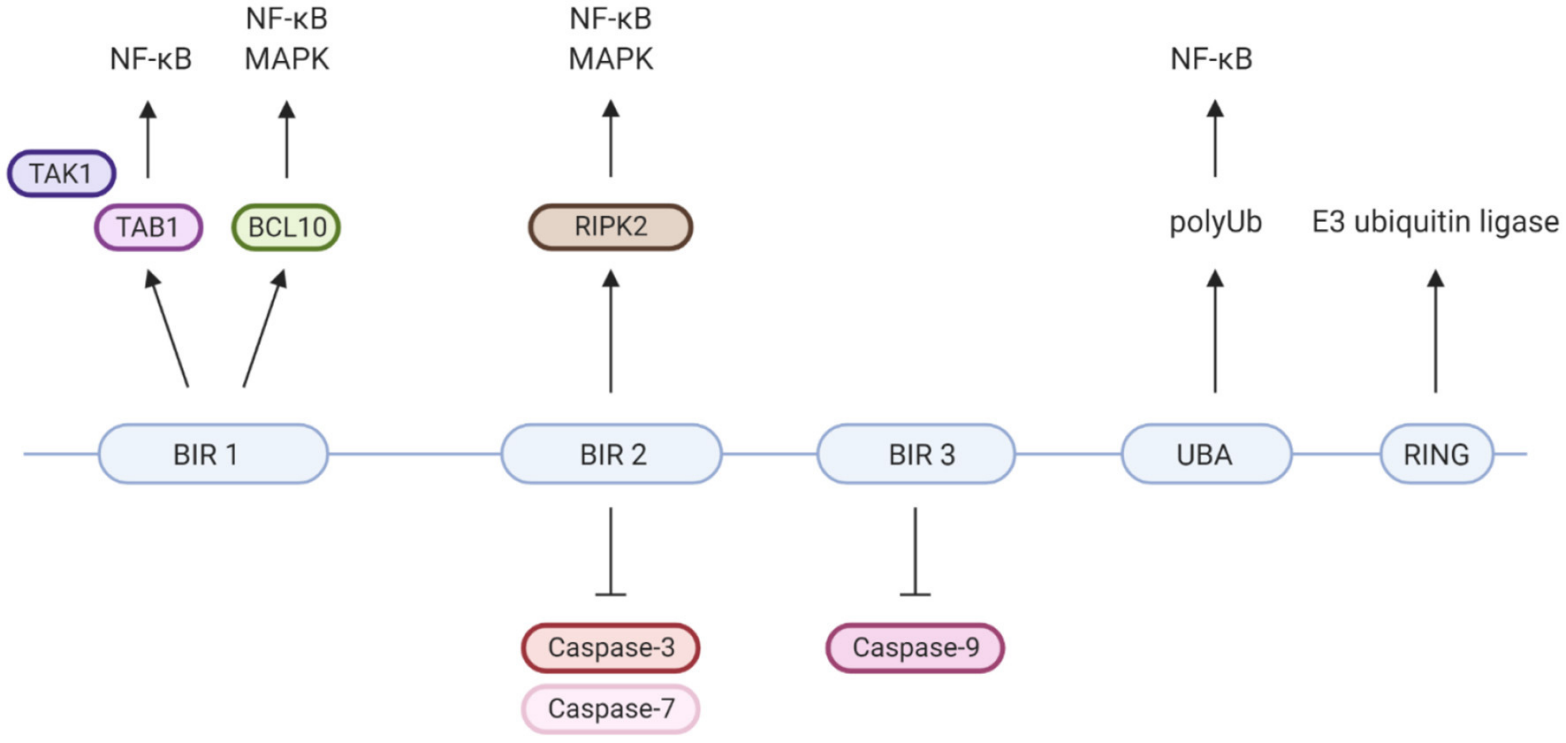
XIAP's protein domains and their individual specific protein interactions and functions. Well-known is that XIAP's BIR2 and 3 domains directly inhibit caspase-3, −7 and −9 (18, 25–27). BIR1 has been isolated as the BCL10-binding region within XIAP. This interaction plays a role in the Dectin-1 signalling pathway (28). Furthermore, BIR1 recruits the TAB1/TAK1 complex, which is essential for activation of the NF-κB and MAPK pathways (29–31). BIR2 interacts with RIPK2, which forms a necessary step in the NOD1/2 signalling pathways leading to NF-κB and MAPK activation (5, 32, 33). The UBA domain is able to directly bind polyubiquitin chains and is required for activation of NF-κB, likely via its binding to polyubiquitinated NEMO (34). The C-terminal RING finger domain of XIAP has E3 ubiquitin ligase function, enabling XIAP to target proteins for proteasomal degradation or alter protein function (35). For example, the ubiquitin ligase activity of XIAP is critical for NOD2 mediated activation of NF-κB, via the polyubiquitination of RIKP2 (36). Created with BioRender.com.

XIAP is a highly conserved, ubiquitously expressed protein belonging to the inhibitor of apoptosis (IAP) family of proteins. It has important structural and functional characteristics in common with cIAP-1 and cIAP-2. The protein is 497 amino acids long and consists of three zinc-binding baculovirus IAP repeat (BIR) domains (hallmarks of IAPs), a ubiquitin-associated (UBA) domain and a really interesting new gene (RING) finger domain (Figure 2) (9, 37). The BIR domains directly inhibit caspase-3, −7 and −9, giving XIAP its anti-apoptotic activity (18, 25–27, 37–39). Besides XIAP's well-known anti-apoptotic properties, more recent studies have shown that XIAP has key functions in immunity (Figure 2). XIAP's BIR domains are involved in non-caspase protein interactions by binding to a specific peptide sequence, the IAP-binding motif (IBM) (35). These interactions mediate XIAP's role in various signalling pathways. The UBA domain can bind directly to polyubiquitin (polyUb) chains, thereby enabling XIAP to participate in Ub-dependent signalling pathways (34). Finally, the RING domain has E3 ubiquitin ligase activity, enabling XIAP to target proteins for proteasomal degradation or alter the activity of modified proteins (35).

## Disease Pathophysiology

**Figure 3:**
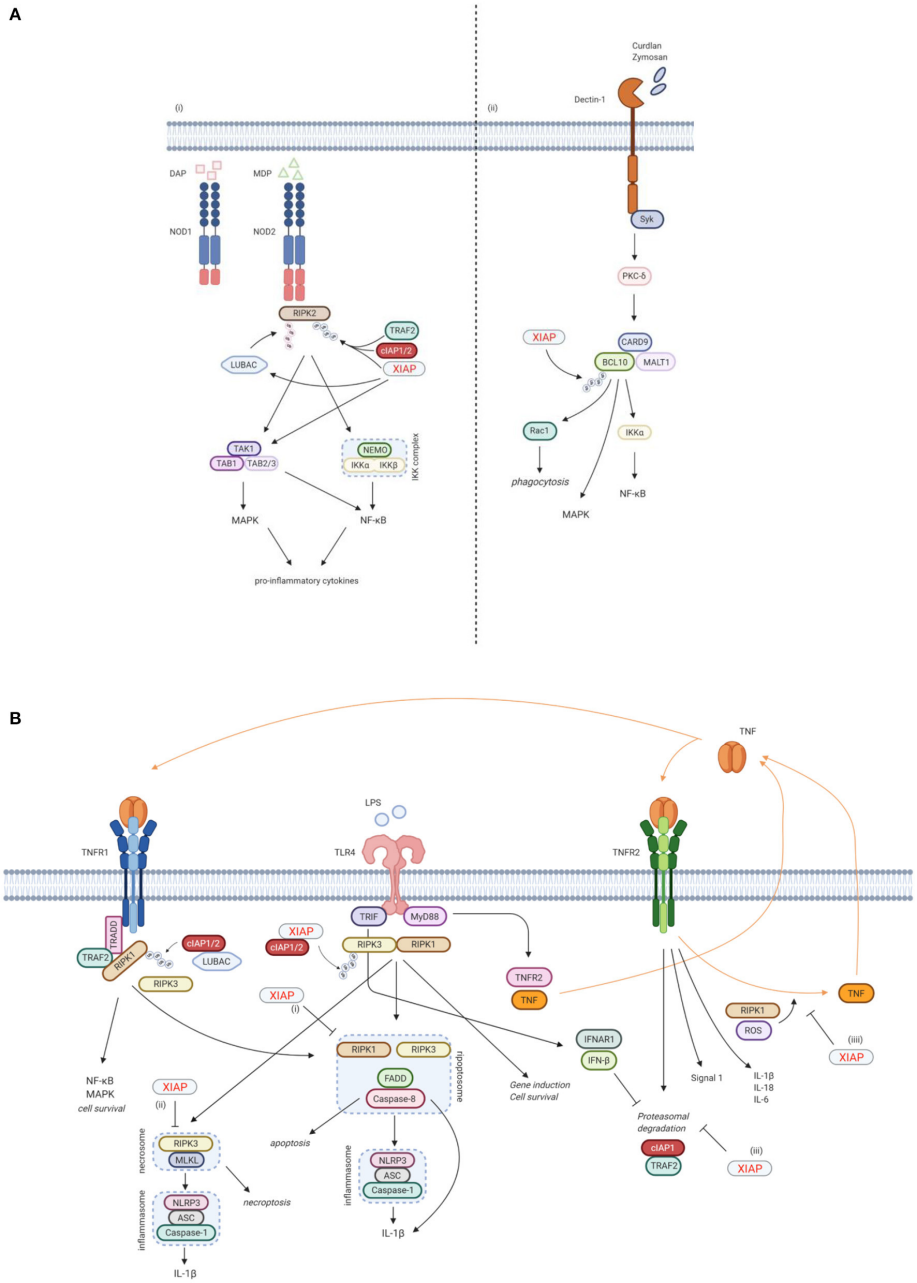
XIAP plays a key function in various immune pathways. **(A)** XIAP is required for pattern recognition receptors (PPR) mediated innate immune responses. (i) XIAP is essential for the NOD1/2 induced activation of the NF-κB and MAPK pathways and secretion of pro-inflammatory cytokines and chemokines (40). NOD1 and NOD2 are intracellular PRRs that bind DAP and MDP, respectively (41, 42). Upon ligand binding, XIAP, together with cIAP1/2, ubiquitinates RIPK2, which subsequently acts as a scaffold for the TAK/TAB1 and IKK complexes, leading to activation of the MAPK and NF-κB pathways, respectively (5, 36, 40). The TAK/TAB1 also links to the IKK complex, thereby inducing activation of the NF-κB pathway (5, 36, 43, 44). Upon NOD activation, XIAP also recruits LUBAC, which in turn conjugates linear Ub chains to RIPK2. The concurrent ubiquitination of RIPK2 by XIAP and LUBAC is necessary for efficient activation of the canonical IKK for NF-κB activation (36, 44, 45). (ii) XIAP is necessary in Dectin-1 signalling. Dectin-1 is a transmembrane PRR that detects β-glucan. Upon Dectin-1 stimulation, XIAP binds and ubiquitinates BCL-10, which is essential for the activation of the NF-κB and MAPK pathways and cytokine production. BCL-10 is also involved in Rac1-dependent phagocytosis, which again relies on ubiquitination of BCL-10 by XIAP (28). **(B)** XIAP regulates the activation of inflammasomes via TLR/TNFR signalling. (i) Following TNFR1 and TLR signalling, RIPK3 can recruit RIKP1 to activate caspase-8 (46, 47). In this process, XIAP has an inhibitory effect on the ripopotosome by controlling the ubiquitination of RIKP1 in a RIPK3-dependent manner (6, 48). (ii) XIAP inhibits MLKL necroptotic signalling, which is promoted by RIPK3 in the absence of caspase-8 (48). (iii) TLR-MyD88 signalling causes the proteasomal degradation of cIAP1 and its adaptor TRAF2 by inducing TNF and TNFR2 signalling. This proteasomal degradation is inhibited by TLR-TRIF induced IFN-β. Loss of XIAP promotes LPS-induced cIAP1 degradation. Subsequently, cIAP1 loss in the absence of XIAP promotes TLR-induced RIPK3 caspase-8 and IL-1β activity, eventually leading to IL-1β maturation, caspase-8 cleavage and enhanced cell death (49). (iiii) TNFR2 activation acts as a signal 1 for priming the canonical inflammasome and induces expression of pro-inflammatory cytokines. XIAP loss and TNFR1 signalling play a role as signal 2 for activation of the inflammasome. This is mediated by RIPK1 and ROS production. In the absence of XIAP, TNFR2 stimulation leads to TNF production, followed by TNFR1 mediated cell death (50). Created with BioRender.com.

**Figure 4:**
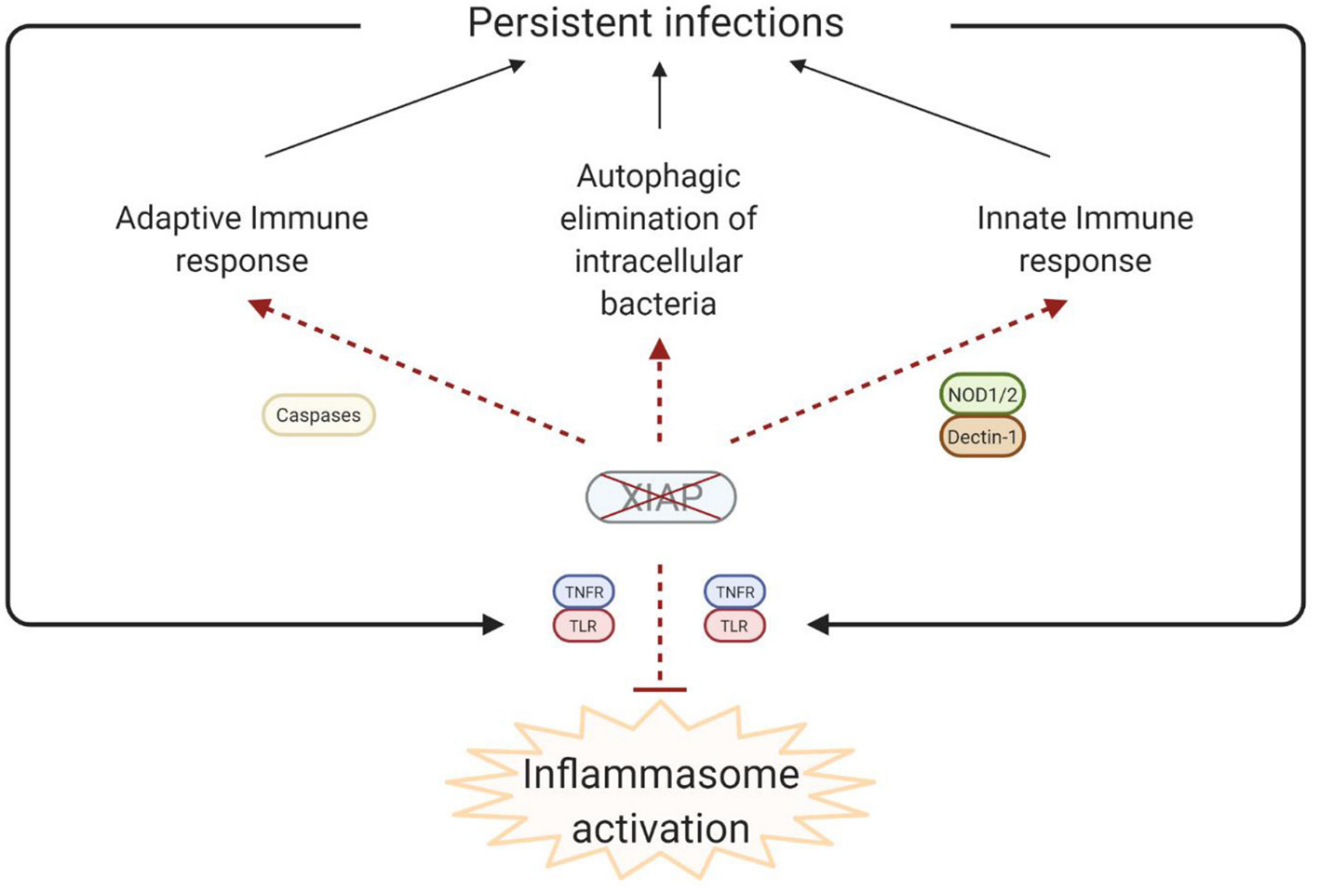
Simplified model of the disease pathophysiology underlying the inflammatory phenomena that are observed in XIAP deficiency. In XIAP deficiency, both the adaptive immune response and the innate immune response are compromised. Additionally, intracellular pathogens are less effectively cleared through xenophagy in the absence of XIAP. Taken together, this results in the persistence of pathogens, which, in turn leads to uncontrolled activation of inflammasomes when regulation by XIAP is lacking. Overall, the result is overproduction of pro-inflammatory cytokines and cell death, leading to a chronic state of hyperinflammation, which can manifest as HLH, IBD, HLH-like disease, arthritis and other inflammatory phenomena. Created with BioRender.com.

Our understanding of the key functions XIAP plays in the immune response (Figures 3, 4) is expanding. Through its anti-apoptotic functions, XIAP is involved in the adaptive immune response. T cells from XIAP deficient patients, particularly invariant natural killer T cells (iNKT) and mucosal-associated invariant T *(*MAIT) cells which express elevated levels of caspases that are inhibited by XIAP, have an increased sensitivity to activation-induced cell death (AICD) (2–4, 51, 52). Thus, it is thought that upon infection, expansion of virus-specific T-cells is suboptimal in XIAP deficient patients.

Besides its anti-apoptotic role however, XIAP is also involved in other signalling pathways that are essential for the innate immune response. Firstly, XIAP is required for NOD1 and NOD2 signalling via the ubiquitination of RIPK2, which results in activation of NF-κB and MAPK pathways and secretion of pro-inflammatory cytokines and chemokines (Figure 3A) (5, 32, 33, 36, 40). NOD-like receptors (NLRs) are intracellular pattern recognition receptors (PRRs) involved in the recognition of degraded products of peptidoglycans from the bacterial cell wall, thereby playing an important role in the innate immune response (5, 36, 40). NOD2, the most studied NLR in relation to XIAP deficiency, is expressed mainly by cells of haematopoietic origin and by Paneth cells in the gut, and is activated by muramyl dipeptide (MDP), a constituent of Gram-negative and Gram-positive bacteria (41, 42). Upon ligand binding, XIAP mediates the ubiquitination of RIPK2 and recruits the linear ubiquitin chain assembly complex (LUBAC) to the NOD receptor, which is essential for NOD2 mediated NF-κB activation (5, 36, 40, 43–45).

Secondly, XIAP is necessary for Dectin-1 signalling (Figure 3A) (28). Dectin-1 is a transmembrane PRR that is involved in antifungal immunity through recognition of β-glucan, a major carbohydrate structure found in fungal cell walls (53). Upon Dectin-1 activation XIAP binds and ubiquitinates BCL10, an essential step for NF-κB and MAPK activation, cytokine production and phagocytosis (28).

Furthermore, XIAP is important in regulating the activation of the NLRP3 inflammasome (Figure 3B). XIAP loss results in dysregulation of classical caspase-1/NLRP3 inflammasome activation, overproduction of pro-inflammatory cytokines and cell death (6, 40, 48, 50, 54–57). First described was the inhibitory effect XIAP has on the ripoptosome, a death-inducing complex comprised of RIKP1, FADD and caspase-8, following both TNFR1 and TLR signalling. This inhibitory effect appears to be mediated by the regulation of RIPK1 ubiquitination by XIAP in a RIPK3-dependent manner (6, 46–48). More recently, XIAP also has been shown to play a role in TNFR2 signalling. In this context, XIAP inhibits the proteasomal degradation of cIAP1 that follows TLR-MyD88 induced TNFR2 signalling. In case of XIAP loss, the degradation of cIAP1 leads to inflammasome activation (49). XIAP also mediates RIPK1 kinase activity and ROS production that occur upon TNFR2 activation, thereby inhibiting the activation and upregulation of the canonical inflammasome (50). It has become clear that the role of XIAP in inflammasome regulation is important, but complex and not yet fully understood. Surprisingly, patients with Cryopyrin-associated periodic syndrome (CAPS), which is caused by a gain-of-function mutations in the *NLRP3* gene, do develop recurrent fever, uveitis and arthritis, similar to XIAP deficient patients, but do not develop HLH, splenomegaly or IBD (1, 58, 59).

Finally, recent studies have shown that XIAP is involved in the autophagic elimination of intracellular bacteria and is required for the efficient fusion of lysosomes with autophagosomes (60, 61). Overall, it is clear that XIAP is important for both the clearance of pathogens and the regulation of the inflammatory response.

It is theorised that in XIAP deficiency there is abnormal persistence of pathogens due to the ineffective immune response, characterised by a decreased cytokine production by myeloid cells and subsequent impaired attraction of neutrophils and phagocytes. Additionally there is uncontrolled inflammasome activation, with overproduction of pro-inflammatory cytokines, and death of lymphocytes and myeloid cells (Figure 4). Overall these processes result in a chronic state of hyperinflammation, which can lead to HLH, IBD, HLH-like disease, arthritis and other inflammatory phenomena.

Most likely, the dysregulation of inflammasome activation plays an important role in the occurrence of HLH in XIAP deficiency (6, 56, 57). Immunisation of mice with alum, an agent that induces an inflammatory response primarily driven by NLRP3 inflammasome activation, caused splenomegaly and elevated splenic infiltration of inflammatory cells, which is reminiscent of the splenomegaly observed in XIAP deficient patients (6). In addition, dysregulation of inflammasome activation causes recurrent HLH, autoinflammation and elevated IL-18 levels in humans that have an activation mutation in the NLRC4 inflammasome (62).

In contrast, it seems that impaired NOD2 signalling is important for the development of IBD in XIAP deficiency. *NOD2* is the most important susceptibility gene for the development of Crohn's disease, indicating the importance of NOD signalling in maintaining intestinal homeostasis (63–65). NOD2 signalling in Paneth cells normally results in secretion of chemokines such as IL-8 and MCP-1. Following impaired signalling, reduced chemoattraction of granulocytes to the gut leads to reduced clearance of pathogens and the chronic granulomatous inflammation that is seen in Crohn's disease (42, 66–72). On the other hand, diminished IL-10 production, which has important anti-inflammatory functions, upon defective NOD2 signalling contributes to the loss of immune regulation (42, 68, 73). This is supported by the fact that IL-10 and IL-10 receptor mutations lead to severe very-early-onset IBD (74). It is likely that other processes also play a role in the aetiology of IBD, such as the impaired NOD1 signalling, diminished xenophagy of intracellular bacteria and the increased susceptibility to apoptosis of MAIT and iNKT cells, which are important cells for the gut immune homeostasis (32, 75, 76). Complete remission of IBD has been described post-HSCT, confirming that the cause most likely involves haematological lineages. Furthermore, XIAP expression was found to be strongly reduced in monocytes from female carriers suffering from IBD, whilst expression in lymphocytes was normal, suggesting a predominant role for myeloid cells (21).

## Clinical Manifestations

Immune dysregulation leads to a range of clinical manifestations in XIAP deficiency including recurrent HLH which is often triggered by EBV infection, IBD, splenomegaly, hypogammaglobulinemia, cytopaenias, and autoinflammatory phenomena (1–3, 21). Table 1 summarises the occurrence of common disease manifestations and their observed frequencies. There may be an intrinsic selection bias in many of the reported case series as a result of the study design. For example, in the paper from Rigaud et al. all patients were first diagnosed with XLP based on their clinical phenotype (2), Aguilar et al. focused their study on patients with colitis (21), while others have focussed on identifying monogenetic causes of early-onset IBD (83, 109), or on patients who have been treated with allogeneic HSCT (77). The clinical phenotype of XIAP deficient patients is highly variable; many different symptoms, that all can occur independently from each other, have been described to date (Tables 1, 2). Patients may suffer from one or multiple symptoms during their disease course. In addition, age of onset of disease is highly variable, with some patients presenting as early as the neonatal period, while others remain symptom free until adulthood. Asymptomatic XIAP deficient males have also been identified by family testing (4, 11).

**Table 1:** Occurrence of common clinical characteristics of XIAP deficiency reported to date.

	**(2)**	**(3)**	**(14)**	**(22)**	**(11)**	**(77)**	**(4)**	**(78)**	**(79)**	**(21)**	**(24)**	**(80)**	**(81)**	**(82)**	**Case reports and small case series (10, 12, 23, 51, 83–108)**	Total^*^
No. of patients	12	10	30	7	9	19	27	10	12	17	6	17	29	7	47	226
No. of families	3	8	11	1	6	NA	17	9	11	11	1	12	19	6	38	
Family History	NA	5 (50)	NA	7 (100)	6 (67)	NA	NA	4 (40)	NA	NA	6 (100)	NA	19 (66)	2 (29)	4	
HLH	11 (92)	9 (90)	22 (73)	0 (0)	6 (67)	16 (84)	10 (37)	10 (100)	7 (58)	7 (41)	1 (17)	11 (65)	23 (79)	3 (43)	30	137 (61%)
EBV-HLH	8 (67)	3 (30)	15 (50)	0 (0)	4 (44)	6 (32)	6 (22)	4 (40)	NA	NA	0	NA	8 (28)	2 (29)	9	56 (25%)
Recurrent HLH or HLH-like illness	6 (50)	6 (60)	20 (67)	1 (14)	5 (56)	NA	NA	8 (80)	5 (42)	NA	1 (17)	10 (59)	NA	2 (29)	19	73 (32%)
Splenomegaly	9 (75)	9 (90)	20 (67)	5 (71)	4 (44)	NA	17 (63)	7 (70)	7 (58)	7 (41)	1 (17)	6 (35)	11 (38)	4 (57)	25	108 (48%)
Cytopenia	NA	9 (90)	NA	3 (43)	6 (67)	NA	NA	8 (80)	1 (8)	NA	1 (17)	1 (6)	13 (45)	2 (29)	21	48 (21%)
Hypogammaglobulinemia	4 (33)	2 (20)	8 (27)	3 (43)	2 (22)	NA	4 (15)	1 (10)	2 (17)	NA	NA	4 (24)	3 (10)	1 (14)	5	32 (14%)
IBD	2 (17)	0	5 (17)	2 (29)	2 (22)	2 (11)	7 (26)	1 (10)	1 (8)	17 (100)	4 (67)	6 (35)	13 (45)	0	14	51 (23%)
Hepatitis	0	0	0	1 (14)	0	0	1 (4)	0	0	0	0	0	NA	0	4	6 (3%)
Uveitis	0	0	0	0	0	0	1 (4)	0	0	0	0	0	NA	0	1	2 (1%)
Arthritis	0	0	0	0	0	0	1 (4)	0	0	1 (6)	1 (17)	0	NA	0	0	2 (1%)
Skin abscesses	0	0	0	0	0	0	1 (4)	0	0	5 (29)	3 (50)	0	NA	0	2	11 (5%)
Recurrent/complicated infections	0	2 (20)	2 (7)	5 (71)	1 (11)	3 (16)	0	0	0	0	3 (50)	0	NA	1 (14)	6	22 (10%)
Age at onset in years(median, range)	3.5	-	4	6	0.5	1	6	1.25	4	7	NED	1.3	3	3.5	-	
	(0.6–22)	(Infancy-8)	(0.1–22)	(1–21)	(0.2–1.7)	(0.2–17)	(0.1–20)	(0.1–3)	(0.25–17)	(0–20)		(0.1–14)	(0.1–17)	(0–5.3)	0–16	
No. of patients with other manifestations	0	0	4 (13)	0	0	2 (11)	5 (19)	0	1 (8)	5 (29)	3 (50)	3 (18)	NA	0	11	
Asymptomatic patients	0	1 (10)	1 (3)	1 (14)	1 (11)	1 (5)	2 (7)	0	1 (8)	0	0	1 (6)	0	1 (14)	5	
Survival	8 (67)	7 (70)	17 (57)	4 (57)	7 (78)	7 (37)	26 (96)	8 (80)	12 (100)	14 (82)	4 (67)	15 (88)	26 (90)	6 (86)	36	

*Numbers indicate the number of patients and, in brackets, the relative frequency in percentage, unless otherwise specified. Data from case reports and small case series with ≤ 5 patients are summarised in one column. NA, not assessed; NED, not enough data*.

* *Some patients may have been reported in more than one publication*.

**Table 2:** Overview of infrequent disease manifestations that have been described in XIAP deficient male patients.

**Non-classical manifestation in XIAP deficiency**		**No. of patients**	**References**
*Gastro-intestinal*	Celiac-like disease	2	(4)
	Eosinophilic colitis	1	(97)
*Hepatic*	Cholangitis	4	(14, 21)
	Granulomatous hepatitis	1	(87)
	Other liver disease	3	(14, 77, 105)
*Renal*	Acute kidney disease	1	(101)
	Renal failure	1	(21)
*Dermatologic*	Erythema Nodosum	2	(4, 24)
	Folliculitis	2	(24, 98)
	Epidermolysis bullosa dystrophica	1	(24)
	Cutaneous Crohn's Disease	1	(83)
*Cardiovascular*	IgA vasculitis with nephritis	1	(98)
	Takayasu arteritis	1	(98)
	Coronary artery dilatation	1	(101)
	Ventricular septal defect	1	(77)
*Haematological*	Coagulopathy	1	(85)
*Pulmonary*	Granulomatous and lymphocytic interstitial lung disease	1	(87)
	Nodular lung disease	1	(77)
	Respiratory failure	1	(91)
*Infectious*	Giardiasis	2	(4)
	Cryptococcosis	1	(14)
	EBV-related pancreatitis	1	(90)
	Persistent urethritis	1	(89)
*Malignancy*	Malignancy	2	(21, 80)
*Neurological*	Facial palsy	1	(4)
	Encephalitis	1	(80)
*Musculoskeletal*	Arthralgia	3	(21, 79, 85)
	JIA	1	(80)
*Other*	Multisystem LCH	1	(103)

More than half of the reported XIAP deficient patients develop HLH. HLH is a life-threatening syndrome characterised by hyperinflammation caused by an uncontrolled and ineffective immune response, in which activated T lymphocytes and macrophages accumulate in organs, and produce high levels of pro-inflammatory cytokines, such as IFN-γ, TNF-α and IL-6, resulting in tissue damage and organ failure (110, 111). The observed high risk for HLH is at least partly related to dysregulation of the NLRP3 inflammasome. Accordingly, patients are known to have chronically elevated IL-18 levels (78, 112). In many cases HLH is triggered by an EBV infection, however patients have also been described to develop HLH in the course of a CMV or HHV6 infection, or in the absence of any clear trigger (1, 4). Given the strong association between HLH and XIAP deficiency, measurement of XIAP expression should be considered at an early stage of evaluation of patients presenting with HLH. Patients presenting with macrophage activation syndrome (MAS) should also be considered for XIAP deficiency testing. There is clinical overlap between HLH and MAS, though the driving pathophysiologic process is thought to differ, but the discussion of the distinctions and the best classification to use for patients with XIAP deficiency is beyond the scope of this review.

In XIAP deficiency, it is not uncommon for patients to suffer from recurrent episodes of HLH, or HLH-like disease. During the latter, patients often have fevers, cytopaenias, splenomegaly or combinations of these, but do not fulfil ≥ 5 of the HLH-2004 diagnostic criteria, or may technically fulfil them but have mild or transient symptoms. This most likely represents attenuated forms of HLH, and in the authors' experience, may be responsive to brief courses of corticosteroids. In support, following splenectomy histopathology revealed haemophagocytosis in the spleen of a XIAP deficient patient (1, 14). Splenomegaly is a classical manifestation of XIAP deficiency, with approximately half of the patients having persistent splenomegaly or experiencing one or more episodes of splenomegaly (4).

Already in the first description of XIAP deficiency Rigaud et al. identified two patients who suffered from colitis (2). Subsequently, Worthey et al. described XIAP deficiency as the underlying disorder in a patient who presented with IBD (51). Today, IBD is a well-recognised manifestation of XIAP deficiency. It strongly resembles Crohn's disease clinically and histologically and is generally severe and refractory to immunosuppressive therapy. Notably, XIAP deficiency can be observed in up to 4% of paediatric-onset IBD and is now considered a Mendelian cause of IBD. Genomic screening for XIAP deficiency and other monogenetic disorders should be considered in paediatric onset or severe and therapy refractory IBD (21, 83, 84, 109, 113, 114). Female carriers of pathologic XIAP mutations can also develop IBD (21, 24). Endoscopy reveals patchy disease, alternating inflammatory lesions with healthy mucosa. All segments of the gastrointestinal tract can be affected, including the stomach, ileum, anus and, in the majority of the cases, the colon (14, 21). Patients may suffer from severe (perianal) fistulae, ulcerations, recurrent strictures and abscesses (4, 14, 21, 51). Infants and young children often present with severe diarrhoea and failure to thrive. Histopathology reveals acute inflammatory lesions, including crypt abscesses, polymorphic infiltrations and epithelioid granulomas in combination with chronic inflammation. Generally, colitis presents at an early age, however, age of onset has been shown to vary widely, ranging from the postnatal period well into adulthood (21, 81). Disease course can be very severe and IBD related mortality in XIAP deficiency has been reported to be relatively high, estimated to be around 10–40%, usually due to gastrointestinal haemorrhage (1, 14). Besides colitis, other gastrointestinal manifestations have also been described in context of XIAP deficiency, including coeliac-like disease characterised by blunted villi and lymphocytic infiltrates, chronic diarrhoea and severe or chronic parasitic infections (4).

Hypogammaglobulinemia, which may be mild and transient, occurs in up to one third of the patients (14, 16). The aetiology of hypogammaglobulinemia in XIAP deficiency seems to differ from that in SAP deficiency. In the latter, a decrease in Ig-isotype switched B-cells is observed, due to a block in germinal centre formation. In contrast, in XIAP deficiency memory B-cell levels are generally normal and the underlying cause of hypogammaglobulinemia is unknown. Various theories on the aetiology have been proposed, including increased AICD of B-cells and the effect of immunosuppressive treatment (14).

Previous reports have shown that ~7% of XIAP deficient patients suffer from other, more rare, inflammatory manifestations (Table 2) (1). With numbers of identified cases increasing, it has become clear that some of these disease manifestations are not uncommon. The importance of recognising these symptoms as possible manifestations of XIAP deficiency should be emphasised. Firstly, hepatitis has been described in various patient cohorts. It is unclear if the liver disease should be considered as an incomplete form of HLH (1, 14). In addition, uveitis and arthritis are now well-recognised manifestations of XIAP deficiency. Both have been described to be recurrent, occurring at a young age and, in the case of uveitis, difficult to treat (4, 21, 24, 85). Some patients with XIAP deficiency also suffer from repeated skin boils and abscesses, or severe acne (4, 24, 86). Besides inflammatory symptoms, XIAP deficient patients regularly suffer from recurrent, prolonged or complicated viral and bacterial infections. These may, but do not necessarily, occur during a period of hypogammaglobulinemia (14).

Finally, asymptomatic XIAP deficient males have been identified in various families, carrying the same XIAP mutation as their symptomatic siblings. Often, absence of XIAP protein and/or XIAP function has been demonstrated in these asymptomatic individuals, once again highlighting the possible importance of other genetic and environmental factors on disease phenotype (4).

## Additional Immunological Findings

No gross abnormalities in the classical immunological parameters and lymphocyte subsets have been reported in asymptomatic or clinically stable XIAP deficient patients. Contrary to what is observed in SAP efficiency, iNKT cell numbers are normal in XIAP patients during wellness (52, 115, 116). T and NK cell cytotoxic responses are normal in XIAP deficiency, which is again in contrast to what is observed in SAP deficiency (11, 12). Serum levels of pro-inflammatory cytokines, including IL-6, IL-2, IFN-γ, TNF-α and neopterin are elevated in XIAP deficient patients with HLH, similar to observations in XLP and FHL patients (78). However, IL-18 levels are found to be greatly elevated during HLH in XIAP deficiency, with significantly higher levels compared to patients with XLP, FHL and EBV-HLH, and levels remain high even during remission (78).

## Diagnosis

The gold standard for diagnosing XIAP deficiency remains the identification of a disease-causing mutation in the *XIAP/BIRC4* gene by genetic sequencing. Ideally, this is complemented by analysis of XIAP expression and functional assays (4). Flow cytometry provides a rapid screening technique in suspected individuals, as the majority of the XIAP deficient patients have decreased or absent XIAP expression (12, 117). Although it must be noted that monoclonal antibodies available for flow cytometry do not recognise XIAP protein truncated before residue 397, while truncated proteins may still retain some cellular functions. Also, patients with a full-length dysfunctional protein may not be identified as XIAP deficient by flow cytometry if missense mutations do not alter the antibody-antigen interaction or protein stability (4). In addition, a functional assay, based on the understanding that XIAP is necessary for NOD2 signalling, can be used for the screening diagnosis of XIAP deficiency; flow cytometric measurement of TNF-α production by monocytes in response to a NOD ligand, such as L18-MDP, can distinguish XIAP deficient cells that have reduced TNF-α production, from wild type cells (21, 51, 79, 83). Flow cytometric studies of XIAP expression or TNF-α production and X-chromosome inactivation profile analysis can also be useful in the evaluation of symptomatic female carriers. Detection of elevated levels of IL-18 in peripheral blood samples can be useful during the evaluation or monitoring of XIAP deficient patients and symptomatic carriers.

## Treatment

Given the large variability in presentation and disease course, there is no general therapeutic recommendation for XIAP deficiency and treatment depends on disease manifestations (3). HLH can be treated according to established protocols (HLH-94 and 2004), which includes corticosteroids and etoposide, though in the authors' experience HLH may be readily controlled with steroid treatment alone (10, 14, 78, 111). Rituximab should be considered in cases of EBV-associated HLH (118). Splenomegaly generally does not require intervention. Only in rare cases of hypersplenism has splenectomy been reported, but recurrent pneumococcal infections may then complicate the clinical course (4). Hypogammaglobulinemia is often transient, but may require treatment with immunoglobulin replacement therapy (11). IBD is initially treated with conventional immunosuppressive agents, including corticosteroids, azathioprine, anti-TNF-α and mesalazine (14, 21, 83, 87). However, as stated previously, IBD is often refractory to immunosuppressive treatment and surgical intervention, including colectomy, abscess drainage and resection of fistulae, is not uncommon (21).

Currently, the only curative treatment option for XIAP deficiency is allogeneic HSCT. In patients with severe disease, including HLH and severe refractory IBD, HSCT may be the treatment of choice. Early reports of HSCT in XIAP deficiency revealed poor outcomes, with long-term survival rates below 50%. Factors associated with an unfavourable outcome were a myeloablative conditioning regimen, with reported survival post-HSCT dropping to 14% in this group, and ongoing HLH at time of transplantation (77). Conditioning regimen related toxicities, such as pulmonary haemorrhage and hepatic veno-occlusive disease, were a common cause of complications early post-HSCT, implying that chemotherapeutics cause increased cytotoxicity in the absence of XIAP (77, 88). Recent reports have shown a more favourable outcome of HSCT following reduced-intensity conditioning (RIC) approaches (1, 88–90, 119). In a larger study Arnold et al. observed a 2-year overall survival of 74% in XIAP deficient HSCT recipients who had been treated with RIC or reduced toxicity conditioning (RTC) regimens, which is similar to reported mortality rates post-HSCT for other forms of HLH [manuscript submitted]. In a Japanese series of patients transplanted with reduced toxicity approaches, 90% survival was observed (81). HLH may complicate the early post-transplantation course, but response to treatment is generally good (78, 81). GvHD was associated with a significantly increased risk of mortality in patients with XIAP deficiency in the study by Arnold et al. [manuscript submitted]. It is hypothesised that the XIAP-deficient tissue environment and the chronic inflammasome activation play a role in the increased risk of GvHD (120, 121). Notably, XIAP-deficient recipient tissues have been shown to predispose to increased GvHD severity and mortality in murine models. Nonetheless, when HSCT is indicated, the combination of a RIC/RTC regimen with a more aggressive approach to GvHD prevention will likely result in lowest mortality and morbidity risks in XIAP deficiency.

## Concluding Remarks

Due to the seriousness of XIAP deficiency and the high-risk nature of curative allogeneic HSCT, it remains important to study novel long-term treatment approaches. New therapeutics may be used to avoid the need for HSCT or as bridging therapy to help improve the patient's clinical condition prior to treatment with curative intent, thereby increasing the chances of a favourable and uncomplicated outcome. Greater understanding of the pathophysiology of XIAP deficiency and the pleiotropic effects that XIAP has on the immune system might create insight into pathways that can be targeted by novel therapeutic agents, such as small molecules. In addition, following the rapid development of gene therapy and editing technologies, lentiviral mediated gene addition or targeted gene correction of the defective *XIAP* gene in autologous haematopoietic stem cells may offer future alternative management options.

## Data Availability Statement

The original contributions presented in the study are included in the article/supplementary materials, further inquiries can be directed to the corresponding author/s.

## Author Contributions

AM wrote the first draught of the manuscript and designed and prepared all figures. CB and RM were responsible for critical revision of the article. All authors contributed to defining the structure and content of the article, manuscript revision, read, and approved the submitted version.

## Conflict of Interest

The authors declare that the research was conducted in the absence of any commercial or financial relationships that could be construed as a potential conflict of interest.

## References

[1] Latour S Aguilar C. XIAP deficiency syndrome in humans. Semin Cell Dev Biol. (2015) 39:115–23. 10.1016/j.semcdb.2015.01.015 25666262

[2] Rigaud S Fondaneche MC Lambert N Pasquier B Mateo V Soulas P. XIAP deficiency in humans causes an X-linked lymphoproliferative syndrome. Nature. (2006) 444:110–4. 10.1038/nature05257 17080092

[3] Marsh RA Madden L Kitchen BJ Mody R McClimon B Jordan MB. XIAP deficiency: a unique primary immunodeficiency best classified as X-linked familial hemophagocytic lymphohistiocytosis and not as X-linked lymphoproliferative disease. Blood. (2010) 116:1079–82. 10.1182/blood-2010-01-256099 20489057 PMC2938130

[4] Speckmann C Lehmberg K Albert MH Damgaard RB Fritsch M Gyrd-Hansen M. X-linked inhibitor of apoptosis (XIAP) deficiency: the spectrum of presenting manifestations beyond hemophagocytic lymphohistiocytosis. Clin Immunol. (2013) 149:133–41. 10.1016/j.clim.2013.07.004 23973892

[5] Damgaard RB Fiil BK Speckmann C Yabal M zur Stadt U Bekker-Jensen S. Disease-causing mutations in the XIAP BIR2 domain impair NOD2-dependent immune signalling. EMBO Mol Med. (2013) 5:1278–95. 10.1002/emmm.201303090 23818254 PMC3944466

[6] Yabal M Muller N Adler H Knies N Gross CJ Damgaard RB. XIAP restricts TNF- and RIP3-dependent cell death and inflammasome activation. Cell Rep. (2014) 7:1796–808. 10.1016/j.celrep.2014.05.008 24882010

[7] Liston P Roy N Tamai K Lefebvre C Baird S Cherton-Horvat G. Suppression of apoptosis in mammalian cells by NAIP and a related family of IAP genes. Nature. (1996) 379:349–53. 10.1038/379349a0 8552191

[8] Uren AG Pakusch M Hawkins CJ Puls KL Vaux DL. Cloning and expression of apoptosis inhibitory protein homologs that function to inhibit apoptosis and/or bind tumor necrosis factor receptor-associated factors. Proc Natl Acad Sci USA. (1996) 93:4974–8. 10.1073/pnas.93.10.4974 8643514 PMC39390

[9] Duckett CS Nava VE Gedrich RW Clem RJ Van Dongen JL Gilfillan MC. A conserved family of cellular genes related to the baculovirus iap gene and encoding apoptosis inhibitors. EMBO J. (1996) 15:2685–94. 10.1002/j.1460-2075.1996.tb00629.x 8654366 PMC450204

[10] Zhao M Kanegane H Ouchi K Imamura T Latour S Miyawaki T. A novel XIAP mutation in a Japanese boy with recurrent pancytopenia and splenomegaly. Haematologica. (2010) 95:688–9. 10.3324/haematol.2009.018010 20015872 PMC2857205

[11] Yang X Kanegane H Nishida N Imamura T Hamamoto K Miyashita R. Clinical and genetic characteristics of XIAP deficiency in Japan. J Clin Immunol. (2012) 32:411–20. 10.1007/s10875-011-9638-z 22228567

[12] Filipovich AH Zhang K Snow AL Marsh RA. X-linked lymphoproliferative syndromes: brothers or distant cousins? Blood. (2010) 116:3398–408. 10.1182/blood-2010-03-275909 20660790 PMC2981470

[13] Seemayer TA Gross TG Egeler RM Pirruccello SJ Davis JR Kelly CM. X-linked lymphoproliferative disease: twenty-five years after the discovery. Pediatr Res. (1995) 38:471–8. 10.1203/00006450-199510000-00001 8559596

[14] Pachlopnik Schmid J Canioni D Moshous D Touzot F Mahlaoui N Hauck F. Clinical similarities and differences of patients with X-linked lymphoproliferative syndrome type 1 (XLP-1/SAP deficiency) versus type 2 (XLP-2/XIAP deficiency). Blood. (2011) 117:1522–9. 10.1182/blood-2010-07-298372 21119115

[15] Booth C Gilmour KC Veys P Gennery AR Slatter MA Chapel H. X-linked lymphoproliferative disease due to SAP/SH2D1A deficiency: a multicenter study on the manifestations, management and outcome of the disease. Blood. (2011) 117:53–62. 10.1182/blood-2010-06-284935 20926771 PMC3374620

[16] Aguilar C Latour S. X-linked inhibitor of apoptosis protein deficiency: more than an X-linked lymphoproliferative syndrome. J Clin Immunol. (2015) 35:331–8. 10.1007/s10875-015-0141-9 25737324

[17] Lopez-Granados E Stacey M Kienzler AK Sierro S Willberg CB Fox CP. A mutation in X-linked inhibitor of apoptosis (G466X) leads to memory inflation of Epstein-Barr virus-specific T cells. Clin Exp Immunol. (2014) 178:470–82. 10.1111/cei.12427 25079909 PMC4238874

[18] Eckelman BP Salvesen GS Scott FL. Human inhibitor of apoptosis proteins: why XIAP is the black sheep of the family. EMBO Rep. (2006) 7:988–94. 10.1038/sj.embor.7400795 17016456 PMC1618369

[19] Pedersen J LaCasse EC Seidelin JB Coskun M Nielsen OH. Inhibitors of apoptosis (IAPs) regulate intestinal immunity and inflammatory bowel disease (IBD) inflammation. Trends Mol Med. (2014) 20:652–65. 10.1016/j.molmed.2014.09.006 25282548

[20] Kim SC. Monozygotic twin cases of XIAP deficiency syndrome. J Pediatr Gastroenterol Nutr. (2018) 67:e101. 10.1097/MPG.0000000000001536 28141680

[21] Aguilar C Lenoir C Lambert N Begue B Brousse N Canioni D. Characterization of Crohn disease in X-linked inhibitor of apoptosis-deficient male patients and female symptomatic carriers. J Allergy Clin Immunol. (2014) 134:1131–41 e9. 10.1016/j.jaci.2014.04.031 24942515

[22] Rigaud S Lopez-Granados E Siberil S Gloire G Lambert N Lenoir C. Human X-linked variable immunodeficiency caused by a hypomorphic mutation in XIAP in association with a rare polymorphism in CD40LG. Blood. (2011) 118:252–61. 10.1182/blood-2011-01-328849 21543760

[23] Yang X Hoshino A Taga T Kunitsu T Ikeda Y Yasumi T. A female patient with incomplete hemophagocytic lymphohistiocytosis caused by a heterozygous XIAP mutation associated with non-random X-chromosome inactivation skewed towards the wild-type XIAP allele. J Clin Immunol. (2015) 35:244–8. 10.1007/s10875-015-0144-6 25744037

[24] Dziadzio M Ammann S Canning C Boyle F Hassan A Cale C. Symptomatic males and female carriers in a large Caucasian kindred with XIAP deficiency. J Clin Immunol. (2015) 35:439–44. 10.1007/s10875-015-0166-0 25943627

[25] Deveraux QL Leo E Stennicke HR Welsh K Salvesen GS Reed JC. Cleavage of human inhibitor of apoptosis protein XIAP results in fragments with distinct specificities for caspases. EMBO J. (1999) 18:5242–51. 10.1093/emboj/18.19.5242 10508158 PMC1171595

[26] Scott FL Denault JB Riedl SJ Shin H Renatus M Salvesen GS. XIAP inhibits caspase-3 and−7 using two binding sites: evolutionarily conserved mechanism of IAPs. EMBO J. (2005) 24:645–55. 10.1038/sj.emboj.7600544 15650747 PMC548652

[27] Shiozaki EN Chai J Rigotti DJ Riedl SJ Li P Srinivasula SM. Mechanism of XIAP-mediated inhibition of caspase-9. Mol Cell. (2003) 11:519–27. 10.1016/S1097-2765(03)00054-6 12620238

[28] Hsieh WC Chuang YT Chiang IH Hsu SC Miaw SC Lai MZ. Inability to resolve specific infection generates innate immunodeficiency syndrome in Xiap-/- mice. Blood. (2014) 124:2847–57. 10.1182/blood-2014-03-564609 25190756

[29] Wang C Deng L Hong M Akkaraju GR Inoue J Chen ZJ. TAK1 is a ubiquitin-dependent kinase of MKK and IKK. Nature. (2001) 412:346–51. 10.1038/35085597 11460167

[30] Lu M Lin SC Huang Y Kang YJ Rich R Lo YC. XIAP induces NF-kappaB activation via the BIR1/TAB1 interaction and BIR1 dimerization. Mol Cell. (2007) 26:689–702. 10.1016/j.molcel.2007.05.006 17560374 PMC1991276

[31] Sanna MG Duckett CS Richter BW Thompson CB Ulevitch RJ. Selective activation of JNK1 is necessary for the anti-apoptotic activity of hILP. Proc Natl Acad Sci USA. (1998) 95:6015–20. 10.1073/pnas.95.11.6015 9600909 PMC27577

[32] Park JH Kim YG Shaw M Kanneganti TD Fujimoto Y Fukase K. Nod1/RICK and TLR signaling regulate chemokine and antimicrobial innate immune responses in mesothelial cells. J Immunol. (2007) 179:514–21. 10.4049/jimmunol.179.1.514 17579072

[33] Yang Y Yin C Pandey A Abbott D Sassetti C Kelliher MA. NOD2 pathway activation by MDP or Mycobacterium tuberculosis infection involves the stable polyubiquitination of Rip2. J Biol Chem. (2007) 282:36223–9. 10.1074/jbc.M703079200 17947236

[34] Gyrd-Hansen M Darding M Miasari M Santoro MM Zender L Xue W. IAPs contain an evolutionarily conserved ubiquitin-binding domain that regulates NF-kappaB as well as cell survival and oncogenesis. Nat Cell Biol. (2008) 10:1309–17. 10.1038/ncb1789 18931663 PMC2818601

[35] Vaux DL Silke J. IAPs, RINGs and ubiquitylation. Nat Rev Mol Cell Biol. (2005) 6:287–97. 10.1038/nrm1621 15803136

[36] Damgaard RB Nachbur U Yabal M Wong WW Fiil BK Kastirr M. The ubiquitin ligase XIAP recruits LUBAC for NOD2 signaling in inflammation and innate immunity. Mol Cell. (2012) 46:746–58. 10.1016/j.molcel.2012.04.014 22607974

[37] Deveraux QL Takahashi R Salvesen GS Reed JC. X-linked IAP is a direct inhibitor of cell-death proteases. Nature. (1997) 388:300–4. 10.1038/40901 9230442

[38] Chai J Shiozaki E Srinivasula SM Wu Q Datta P Alnemri ES. Structural basis of caspase-7 inhibition by XIAP. Cell. (2001) 104:769–80. 10.1016/S0092-8674(01)00272-0 11257230

[39] Huang Y Park YC Rich RL Segal D Myszka DG Wu H. Structural basis of caspase inhibition by XIAP: differential roles of the linker versus the BIR domain. Cell. (2001) 104:781–90. 10.1016/S0092-8674(02)02075-5 11257231

[40] Bertrand MJ Doiron K Labbe K Korneluk RG Barker PA Saleh M. Cellular inhibitors of apoptosis cIAP1 and cIAP2 are required for innate immunity signaling by the pattern recognition receptors NOD1 and NOD2. Immunity. (2009) 30:789–801. 10.1016/j.immuni.2009.04.011 19464198

[41] Philpott DJ Sorbara MT Robertson SJ Croitoru K Girardin SE. NOD proteins: regulators of inflammation in health and disease. Nat Rev Immunol. (2014) 14:9–23. 10.1038/nri3565 24336102

[42] Tattoli I Travassos LH Carneiro LA Magalhaes JG Girardin SE. The Nodosome: Nod1 and Nod2 control bacterial infections and inflammation. Semin Immunopathol. (2007) 29:289–301. 10.1007/s00281-007-0083-2 17690884

[43] Hasegawa M Fujimoto Y Lucas PC Nakano H Fukase K Nunez G. A critical role of RICK/RIP2 polyubiquitination in Nod-induced NF-kappaB activation. EMBO J. (2008) 27:373–83. 10.1038/sj.emboj.7601962 18079694 PMC2234345

[44] Saxena M Yeretssian G. NOD-Like Receptors: Master Regulators of Inflammation and Cancer. Front Immunol. (2014) 5:327. 10.3389/fimmu.2014.00327 25071785 PMC4095565

[45] Tokunaga F Iwai K. Linear ubiquitination: a novel NF-kappaB regulatory mechanism for inflammatory and immune responses by the LUBAC ubiquitin ligase complex. Endocr J. (2012) 59:641–52. 10.1507/endocrj.EJ12-0148 22673407

[46] Mandal P Berger SB Pillay S Moriwaki K Huang C Guo H. RIP3 induces apoptosis independent of pronecrotic kinase activity. Mol Cell. (2014) 56:481–95. 10.1016/j.molcel.2014.10.021 25459880 PMC4512186

[47] Newton K Dugger DL Wickliffe KE Kapoor N de Almagro MC Vucic D. Activity of protein kinase RIPK3 determines whether cells die by necroptosis or apoptosis. Science. (2014) 343:1357–60. 10.1126/science.1249361 24557836

[48] Lawlor KE Khan N Mildenhall A Gerlic M Croker BA D'Cruz AA. RIPK3 promotes cell death and NLRP3 inflammasome activation in the absence of MLKL. Nat Commun. (2015) 6:6282. 10.1038/ncomms7282 25693118 PMC4346630

[49] Lawlor KE Feltham R Yabal M Conos SA Chen KW Ziehe S. XIAP Loss Triggers RIPK3- and Caspase-8-Driven IL-1beta activation and cell death as a consequence of TLR-MyD88-induced cIAP1-TRAF2 degradation. Cell Rep. (2017) 20:668–82. 10.1016/j.celrep.2017.06.073 28723569

[50] Knop J Spilgies LM Rufli S Reinhart R Vasilikos L Yabal M. TNFR2 induced priming of the inflammasome leads to a RIPK1-dependent cell death in the absence of XIAP. Cell Death Dis. (2019) 10:700. 10.1038/s41419-019-1938-x 31541082 PMC6754467

[51] Worthey EA Mayer AN Syverson GD Helbling D Bonacci BB Decker B. Making a definitive diagnosis: successful clinical application of whole exome sequencing in a child with intractable inflammatory bowel disease. Genet Med. (2011) 13:255–62. 10.1097/GIM.0b013e3182088158 21173700

[52] Gerart S Siberil S Martin E Lenoir C Aguilar C Picard C. Human iNKT and MAIT cells exhibit a PLZF-dependent proapoptotic propensity that is counterbalanced by XIAP. Blood. (2013) 121:614–23. 10.1182/blood-2012-09-456095 23223428 PMC3824284

[53] Hardison SE Brown GD. C-type lectin receptors orchestrate antifungal immunity. Nat Immunol. (2012) 13:817–22. 10.1038/ni.2369 22910394 PMC3432564

[54] Moulin M Anderton H Voss AK Thomas T Wong WW Bankovacki A. IAPs limit activation of RIP kinases by TNF receptor 1 during development. EMBO J. (2012) 31:1679–91. 10.1038/emboj.2012.18 22327219 PMC3321198

[55] Vanden Berghe T Linkermann A Jouan-Lanhouet S Walczak H Vandenabeele P. Regulated necrosis: the expanding network of non-apoptotic cell death pathways. Nat Rev Mol Cell Biol. (2014) 15:135–47. 10.1038/nrm3737 24452471

[56] Vince JE Wong WW Gentle I Lawlor KE Allam R O'Reilly L. Inhibitor of apoptosis proteins limit RIP3 kinase-dependent interleukin-1 activation. Immunity. (2012) 36:215–27. 10.1016/j.immuni.2012.01.012 22365665

[57] Wong WW Vince JE Lalaoui N Lawlor KE Chau D Bankovacki A. cIAPs and XIAP regulate myelopoiesis through cytokine production in an RIPK1- and RIPK3-dependent manner. Blood. (2014) 123:2562–72. 10.1182/blood-2013-06-510743 24497535

[58] Yu JR Leslie KS. Cryopyrin-associated periodic syndrome: an update on diagnosis and treatment response. Curr Allergy Asthma Rep. (2011) 11:12–20. 10.1007/s11882-010-0160-9 21104172 PMC3020304

[59] Levy R Gerard L Kuemmerle-Deschner J Lachmann HJ Kone-Paut I Cantarini L. Phenotypic and genotypic characteristics of cryopyrin-associated periodic syndrome: a series of 136 patients from the Eurofever Registry. Ann Rheum Dis. (2015) 74:2043–9. 10.1136/annrheumdis-2013-204991 25038238

[60] Schwerd T Pandey S Yang HT Bagola K Jameson E Jung J. Impaired antibacterial autophagy links granulomatous intestinal inflammation in Niemann-Pick disease type C1 and XIAP deficiency with NOD2 variants in Crohn's disease. Gut. (2017) 66:1060–73. 10.1136/gutjnl-2015-310382 26953272 PMC5532464

[61] Gradzka S Thomas OS Kretz O Haimovici A Vasilikos L Wong WW. Inhibitor of apoptosis proteins are required for effective fusion of autophagosomes with lysosomes. Cell Death Dis. (2018) 9:529. 10.1038/s41419-018-0508-y 29743550 PMC5943300

[62] Romberg N Al Moussawi K Nelson-Williams C Stiegler AL Loring E Choi M. Mutation of NLRC4 causes a syndrome of enterocolitis and autoinflammation. Nat Genet. (2014) 46:1135–9. 10.1038/ng.3066 25217960 PMC4177367

[63] Hugot JP Chamaillard M Zouali H Lesage S Cezard JP Belaiche J. Association of NOD2 leucine-rich repeat variants with susceptibility to Crohn's disease. Nature. (2001) 411:599–603. 10.1038/35079107 11385576

[64] Ogura Y Bonen DK Inohara N Nicolae DL Chen FF Ramos R. A frameshift mutation in NOD2 associated with susceptibility to Crohn's disease. Nature. (2001) 411:603–6. 10.1038/35079114 11385577

[65] Strober W Asano N Fuss I Kitani A Watanabe T. Cellular and molecular mechanisms underlying NOD2 risk-associated polymorphisms in Crohn's disease. Immunol Rev. (2014) 260:249–60. 10.1111/imr.12193 24942694

[66] Marks DJ Harbord MW MacAllister R Rahman FZ Young J Al-Lazikani B. Defective acute inflammation in Crohn's disease: a clinical investigation. Lancet. (2006) 367:668–78. 10.1016/S0140-6736(06)68265-2 16503465

[67] Smith AM Rahman FZ Hayee B Graham SJ Marks DJ Sewell GW. Disordered macrophage cytokine secretion underlies impaired acute inflammation and bacterial clearance in Crohn's disease. J Exp Med. (2009) 206:1883–97. 10.1084/jem.20091233 19652016 PMC2737162

[68] Casanova JL Abel L. Revisiting Crohn's disease as a primary immunodeficiency of macrophages. J Exp Med. (2009) 206:1839–43. 10.1084/jem.20091683 19687225 PMC2737171

[69] Kobayashi KS Chamaillard M Ogura Y Henegariu O Inohara N Nunez G. Nod2-dependent regulation of innate and adaptive immunity in the intestinal tract. Science. (2005) 307:731–4. 10.1126/science.1104911 15692051

[70] Lala S Ogura Y Osborne C Hor SY Bromfield A Davies S. Crohn's disease and the NOD2 gene: a role for paneth cells. Gastroenterology. (2003) 125:47–57. 10.1016/S0016-5085(03)00661-9 12851870

[71] Ogura Y Lala S Xin W Smith E Dowds TA Chen FF. Expression of NOD2 in Paneth cells: a possible link to Crohn's ileitis. Gut. (2003) 52:1591–7. 10.1136/gut.52.11.1591 14570728 PMC1773866

[72] Wehkamp J Harder J Weichenthal M Schwab M Schaffeler E Schlee M. NOD2 (CARD15) mutations in Crohn's disease are associated with diminished mucosal alpha-defensin expression. Gut. (2004) 53:1658–64. 10.1136/gut.2003.032805 15479689 PMC1774270

[73] Netea MG Kullberg BJ de Jong DJ Franke B Sprong T Naber TH. NOD2 mediates anti-inflammatory signals induced by TLR2 ligands: implications for Crohn's disease. Eur J Immunol. (2004) 34:2052–9. 10.1002/eji.200425229 15214053

[74] Glocker EO Kotlarz D Klein C Shah N Grimbacher B. IL-10 and IL-10 receptor defects in humans. Ann N Y Acad Sci. (2011) 1246:102–7. 10.1111/j.1749-6632.2011.06339.x 22236434

[75] Serriari NE Eoche M Lamotte L Lion J Fumery M Marcelo P. Innate mucosal-associated invariant T (MAIT) cells are activated in inflammatory bowel diseases. Clin Exp Immunol. (2014) 176:266–74. 10.1111/cei.12277 24450998 PMC3992039

[76] Olszak T Neves JF Dowds CM Baker K Glickman J Davidson NO. Protective mucosal immunity mediated by epithelial CD1d and IL-10. Nature. (2014) 509:497–502. 10.1038/nature13150 24717441 PMC4132962

[77] Marsh RA Rao K Satwani P Lehmberg K Muller I Li D. Allogeneic hematopoietic cell transplantation for XIAP deficiency: an international survey reveals poor outcomes. Blood. (2013) 121:877–83. 10.1182/blood-2012-06-432500 23131490 PMC5162550

[78] Wada T Kanegane H Ohta K Katoh F Imamura T Nakazawa Y. Sustained elevation of serum interleukin-18 and its association with hemophagocytic lymphohistiocytosis in XIAP deficiency. Cytokine. (2014) 65:74–8. 10.1016/j.cyto.2013.09.007 24084330

[79] Ammann S Elling R Gyrd-Hansen M Duckers G Bredius R Burns SO. A new functional assay for the diagnosis of X-linked inhibitor of apoptosis (XIAP) deficiency. Clin Exp Immunol. (2014) 176:394–400. 10.1111/cei.12306 24611904 PMC4008984

[80] Nishida N Yang X Takasaki I Imai K Kato K Inoue Y. Dysgammaglobulinemia associated with Glu349del, a hypomorphic XIAP mutation. J Invest Allerg Clin. (2015) 25:205–13. 26182687

[81] Ono S Okano T Hoshino A Yanagimachi M Hamamoto K Nakazawa Y. Hematopoietic stem cell transplantation for XIAP deficiency in Japan. J Clin Immunol. (2017) 37:85–91. 10.1007/s10875-016-0348-4 27815752 PMC7101905

[82] Xu T Zhao Q Li W Chen X Xue X Chen Z. X-linked lymphoproliferative syndrome in mainland China: review of clinical, genetic, and immunological characteristic. Eur J Pediatr. (2020) 179:327–38. 10.1007/s00431-019-03512-7 31754776 PMC6970958

[83] Zeissig Y Petersen BS Milutinovic S Bosse E Mayr G Peuker K. XIAP variants in male Crohn's disease. Gut. (2015) 64:66–76. 10.1136/gutjnl-2013-306520 24572142

[84] Fang YH Luo YY Yu JD Lou JG Chen J. Phenotypic and genotypic characterization of inflammatory bowel disease in children under six years of age in China. World J Gastroenterol. (2018) 24:1035–45. 10.3748/wjg.v24.i9.1035 29531467 PMC5840468

[85] Basiaga ML Weiss PF Behrens EM. BIRC4 Mutation: an important rare cause of uveitis. J Clin Rheumatol. (2015) 21:444–7. 10.1097/RHU.0000000000000327 26513308 PMC4654974

[86] Girardelli M Arrigo S Barabino A Loganes C Morreale G Crovella S. The diagnostic challenge of very early-onset enterocolitis in an infant with XIAP deficiency. BMC Pediatr. (2015) 15:208. 10.1186/s12887-015-0522-5 26671016 PMC4678727

[87] Steele CL Dore M Ammann S Loughrey M Montero A Burns SO. X-linked inhibitor of apoptosis complicated by Granulomatous Lymphocytic Interstitial Lung Disease (GLILD) and granulomatous hepatitis. J Clin Immunol. (2016) 36:733–8. 10.1007/s10875-016-0320-3 27492372

[88] Worth AJ Nikolajeva O Chiesa R Rao K Veys P Amrolia PJ. Successful stem cell transplant with antibody-based conditioning for XIAP deficiency with refractory hemophagocytic lymphohistiocytosis. Blood. (2013) 121:4966–8. 10.1182/blood-2013-01-478735 23766462

[89] Varghese AS Lee H Bonney D Hughes S Wynn R. Complications of reduced intensity conditioning HSCT for XIAP deficiency (Alloimmune Cytopenias and HLH) successfully managed with donor lymphocyte infusion. J Pediatr Hematol Oncol. (2015) 37:e198–9. 10.1097/MPH.0000000000000191 24942029

[90] Yang J Zhu GH Wang B Zhang R Jia CG Yan Y. Haploidentical hematopoietic stem cell transplantation for XIAP deficiency: a single-center report. J Clin Immunol. (2020) 40:893–900. 10.1007/s10875-020-00795-6 32627096

[91] Lekbua A Ouahed J O'Connell AE Kahn SA Goldsmith JD Imamura T. Risk-factors associated with poor outcomes in VEO-IBD secondary to XIAP deficiency: a case report and literature review. J Pediatr Gastroenterol Nutr. (2019) 69:e13–8. 10.1097/MPG.0000000000002297 31232887 PMC6607918

[92] Christiansen M Ammann S Speckmann C Mogensen TH. XIAP deficiency and MEFV variants resulting in an autoinflammatory lymphoproliferative syndrome. BMJ Case Rep. (2016) 2016:bcr2016216922. 10.26226/morressier.57bc1755d462b80290b4d803 27681353 PMC5051366

[93] Jiang MY Guo X Sun SW Li Q Zhu YP. Successful allogeneic hematopoietic stem cell transplantation in a boy with X-linked inhibitor of apoptosis deficiency presenting with hemophagocytic lymphohistiocytosis: a case report. Exp Ther Med. (2016) 12:1341–4. 10.3892/etm.2016.3498 27602064 PMC4998177

[94] Chellapandian D Krueger J Schechter T Gassas A Weitzman S Naqvi A. Successful allogeneic hematopoietic stem cell transplantation in XIAP deficiency using reduced-intensity conditioning. Pediatr Blood Cancer. (2016) 63:355–7. 10.1002/pbc.25756 26398727

[95] Inoue K Miura H Hoshino A Kamiya T Tanita K Ohye T. Inherited chromosomally integrated human herpesvirus-6 in a patient with XIAP deficiency. Transpl Infect Dis. (2020) 2020:e13331. 10.1111/tid.13331 32424944

[96] Vieth S Ammann S Schwarz K Hartel C Schultz C Lehmberg K. Clinical phenotype and functional analysis of a rare XIAP/BIRC4 mutation. Klin Padiatr. (2013) 225:343–6. 10.1055/s-0033-1355393 24166087

[97] Tang J Zhou X Wang L Hu G Zheng B Wang C. Eosinophilic colitis in a boy with a novel XIAP mutation: a case report. BMC Pediatr. (2020) 20:171. 10.1186/s12887-020-02075-z 32305064 PMC7165398

[98] Takeuchi I Kawai T Nambu M Migita O Yoshimura S Nishimura K. X-linked inhibitor of apoptosis protein deficiency complicated with Crohn's disease-like enterocolitis and Takayasu arteritis: a case report. Clin Immunol. (2020) 217:108495. 10.1016/j.clim.2020.108495 32540394

[99] O'Rafferty C Velangi M Lawson S Hiwarkar P Motwani J. IFN Block, Treosulfan conditioning and alphabeta T cell deplete PBSCT for XIAP-deficient HLH. J Clin Immunol. (2017) 37:511–3. 10.1007/s10875-017-0413-7 28639166

[100] Kelsen JR Dawany N Martinez A Grochowski CM Maurer K Rappaport E. A *de novo* whole gene deletion of XIAP detected by exome sequencing analysis in very early onset inflammatory bowel disease: a case report. BMC Gastroenterol. (2015) 15:160. 10.1186/s12876-015-0394-z 26581487 PMC4652404

[101] Chen RY Li XZ Lin Q Zhu Y Shen YY Xu QY. Epstein-Barr virus-related hemophagocytic lymphohistiocytosis complicated with coronary artery dilation and acute renal injury in a boy with a novel X-linked inhibitor of apoptosis protein (XIAP) variant: a case report. BMC Pediatr. (2020) 20:456. 10.1186/s12887-020-02359-4 33008347 PMC7531141

[102] Beser OF Cokugras FC Kutlu T Erginoz E Gulcu D Kasapcopur O. Association of familial Mediterranean fever in Turkish children with inflammatory bowel disease. Turk Pediatri Ars. (2014) 49:198–202. 10.5152/tpa.2014.1998 26078663 PMC4462301

[103] Guo X Li Q Gao J. Langerhans cell histiocytosis complicated with hemophagocytic lymphohistiocytosis in a boy with a novel XIAP mutation: a case report. Medicine (Baltimore). (2018) 97:e13019. 10.1097/MD.0000000000013019 30383659 PMC6221634

[104] Jin YY Zhou W Tian ZQ Chen TX. Variable clinical phenotypes of X-linked lymphoproliferative syndrome in China: report of five cases with three novel mutations and review of the literature. Hum Immunol. (2016) 77:658–66. 10.1016/j.humimm.2016.06.005 27288720

[105] Sun J Ying W Liu D Hui X Yu Y Wang J. Clinical and genetic features of 5 Chinese patients with X-linked lymphoproliferative syndrome. Scand J Immunol. (2013) 78:463–7. 10.1111/sji.12103 23944711

[106] Zhong Y Huang CH Soe WM Chan KW Isa MS Soh J. A Novel X-Linked inhibitor of apoptosis deficient variant showing attenuated epstein-barr virus response. J Pediatric Infect Dis Soc. (2020) 10:345–8. 10.1093/jpids/piaa048 32448891

[107] Horn PC Belohradsky BH Urban C Weber-Mzell D Meindl A Schuster V. Two new families with X-linked inhibitor of apoptosis deficiency and a review of all 26 published cases. J Allergy Clin Immunol. (2011) 127:544–6. 10.1016/j.jaci.2010.11.040 21281876

[108] Shabani M Razaghian A Alimadadi H Shiari R Shahrooei M Parvaneh N. Different phenotypes of the same XIAP mutation in a family: a case of XIAP deficiency with juvenile idiopathic arthritis. Pediatr Blood Cancer. (2019) 66:e27593. 10.1002/pbc.27593 30604482

[109] Uhlig HH Schwerd T Koletzko S Shah N Kammermeier J Elkadri A. The diagnostic approach to monogenic very early onset inflammatory bowel disease. Gastroenterology. (2014) 147:990–1007 e3. 10.1053/j.gastro.2014.07.023 25058236 PMC5376484

[110] Usmani GN Woda BA Newburger PE. Advances in understanding the pathogenesis of HLH. Br J Haematol. (2013) 161:609–22. 10.1111/bjh.12293 23577835

[111] Henter JI Horne A Arico M Egeler RM Filipovich AH Imashuku S. HLH-2004: Diagnostic and therapeutic guidelines for hemophagocytic lymphohistiocytosis. Pediatr Blood Cancer. (2007) 48:124–31. 10.1002/pbc.21039 16937360

[112] van de Veerdonk FL Netea MG Dinarello CA Joosten LA. Inflammasome activation and IL-1beta and IL-18 processing during infection. Trends Immunol. (2011) 32:110–6. 10.1016/j.it.2011.01.003 21333600

[113] Speckmann C Ehl S. XIAP deficiency is a mendelian cause of late-onset IBD. Gut. (2014) 63:1031–2. 10.1136/gutjnl-2013-306474 24326742

[114] Quaranta M Wilson R Goncalves Serra E Pandey S Schwerd T Gilmour K. Consequences of Identifying XIAP deficiency in an adult patient with inflammatory bowel disease. Gastroenterology. (2018) 155:231–4. 10.1053/j.gastro.2018.03.069 29894681

[115] Marsh RA Villanueva J Kim MO Zhang K Marmer D Risma KA. Patients with X-linked lymphoproliferative disease due to BIRC4 mutation have normal invariant natural killer T-cell populations. Clin Immunol. (2009) 132:116–23. 10.1016/j.clim.2009.03.517 19398375 PMC2729708

[116] Bauler LD Duckett CS O'Riordan MX. XIAP regulates cytosol-specific innate immunity to Listeria infection. PLoS Pathog. (2008) 4:e1000142. 10.1371/journal.ppat.1000142 18769721 PMC2516935

[117] Marsh RA Villanueva J Zhang K Snow AL Su HC Madden L. A rapid flow cytometric screening test for X-linked lymphoproliferative disease due to XIAP deficiency. Cytometry B Clin Cytom. (2009) 76:334–44. 10.1002/cyto.b.20473 19288545 PMC2728163

[118] Chellapandian D Das R Zelley K Wiener SJ Zhao H Teachey DT. Treatment of Epstein Barr virus-induced haemophagocytic lymphohistiocytosis with rituximab-containing chemo-immunotherapeutic regimens. Br J Haematol. (2013) 162:376–82. 10.1111/bjh.12386 23692048 PMC3776423

[119] Tsuma Y Imamura T Ichise E Sakamoto K Ouchi K Osone S. Successful treatment of idiopathic colitis related to XIAP deficiency with allo-HSCT using reduced-intensity conditioning. Pediatr Transplant. (2015) 19:E25–8. 10.1111/petr.12405 25412586

[120] Toubai T Rossi C Oravecz-Wilson K Liu C Zajac C Wu SJ. IAPs protect host target tissues from graft-versus-host disease in mice. Blood Adv. (2017) 1:1517–32. 10.1182/bloodadvances.2017004242 29296793 PMC5728459

[121] Muller N Fischer JC Yabal M Haas T Poeck H Jost PJ. XIAP deficiency in hematopoietic recipient cells drives donor T-cell activation and GvHD in mice. Eur J Immunol. (2019) 49:504–7. 10.1002/eji.201847818 30585320

